# Association of benzodiazepines, opioids and tricyclic antidepressants use and falls in trauma patients: Conditional effect of age

**DOI:** 10.1371/journal.pone.0227696

**Published:** 2020-01-15

**Authors:** Sergio Cordovilla-Guardia, Tania Bautista Molina, Cristina Franco-Antonio, Esperanza Santano-Mogena, Raquel Vilar-López

**Affiliations:** 1 Nursing Department, Nursing and Occupational Therapy College, University of Extremadura, Cáceres, Spain; 2 Health and Care Research Group (GISyC), University of Extremadura, Cáceres, Spain; 3 Hospital Universitario Virgen de las Nieves, Granada, Spain; 4 Mind, Brain and Behavior Research Centre, University of Granada, Granada, Spain; 5 Andalusian Observatory on Drugs and Addictions, Granada, Spain; University of Minnesota, UNITED STATES

## Abstract

**Introduction:**

The relationship between benzodiazepines, opioids and tricyclic antidepressants and trauma is of great importance because of increased consumption and the growing evidence of a positive association among older adults. The objective of this study was to determine the effect size of the association between the consumption of psychotropic medications /opioids and falls in patients who have suffered trauma by studying the role of other variables in this relationship.

**Method:**

From 2011 to 2016, the presence of benzodiazepines, opioids and tricyclic antidepressants and other drugs in 1060 patients admitted for trauma at a level I trauma hospital was analysed. Multivariate models were used to measure the adjusted effect size of the association between consumption of benzodiazepines, opioids and tricyclic antidepressants and falls, and the effect of age on this association was studied.

**Results:**

A total of 192 patients tested positive for benzodiazepines, opioids and tricyclic antidepressants, with same-level falls being the most frequent mechanism of injury in this group (40.1%), with an odds ratio of 1.96 (1.40–2.75), *p < 0*.*001*. Once other covariates were introduced, this association was not observed, leaving only age, gender (woman) and, to a lesser extent, sensory conditions as variables associated with falls. Age acted as an effect modifier between benzodiazepines, opioids and tricyclic antidepressants and falls, with significant effect sizes starting at 51.9 years of age.

**Conclusions:**

The association between the consumption of benzodiazepines, opioids and tricyclic antidepressants and falls in patients admitted for trauma is conditioned by other confounding variables, with age being the most influential confounding variable.

## Introduction

The abusive consumption of substances such as alcohol, cannabis or cocaine poses a substantial morbidity and mortality burden [1]. However, the consumption of benzodiazepines, opioids and tricyclic antidepressants garnering attention because the consumption of these medications, with or without a prescription, has increased considerably in recent years [2–4]. And although there is a worrying increase in consumption in adolescents [5], the consumption of these substances is still higher in women and older adults [2,3,6]. The increase in average life expectancy, social changes such as increased stress, the increased number of unemployed people, changes in family roles or an over diagnosis and excessive treatment could be a few reasons for this increase [3,4,7–9].

While the short-term therapeutic efficacy of benzodiazepines, opioids and tricyclic antidepressants is not in doubt, there are risks associated with their use. In this sense, the association found between the consumption of benzodiazepines and falls and fractures is especially noteworthy [10–12], as is its association with increased risk of traffic accidents and injuries [13,14]. Some authors have found that doses of diazepam of 3 mg/day or more increase the risk of hip fracture by 50%, which increases to 80% if the treatment lasts more than 1 month [11]. The risk of psychotropic medication-related trauma is aggravated in elderly patients [11], with an increased risk of falls, osteoporosis and fractures [15–17].

Falls are one of the most important causes of mortality and disability among older people [16]. In addition, falls lead to numerous hospital admissions, representing almost 90% of all fractures [16]. One-third of people over 65 experience one or more episodes of falls every year [17], whose most common sequelae are pain, functional limitations, disability, long-term care and death, in many cases [18]. Even those falls that do not lead to any injury usually begin a cycle of fear that leads to limited activities of daily living, occasionally losing independence in the basic activities of self-care [17].

A high prevalence of traumatic episodes highlights the need to investigate its causes, which may guide us towards possible intervention strategies [17,19,20]. Although numerous studies have highlighted the association between the consumption of benzodiazepines, opioids and tricyclic antidepressants and falls/fractures [10,11,20], several variables could be confounding this relationship. On the one hand, it is known that the abusive use of some psychotropic medications can affect bone density, leading to osteoporosis, fractures and falls [21]. On the other hand, the consumption of benzodiazepines, opioids and tricyclic antidepressants is linked to an increase in body mass index (BMI) [16–18]. There is evidence that obesity may play an important role in falls due to lower levels of physical activity, higher levels of pain or problems with postural balance [20,21]. In overweight or obese people who suffer a same-level fall, the impact force is greater; therefore, the consequence of a fall will be proportionally higher [22]. On the other hand, cognitive impairment, even mild, and sensory conditions, such as cataracts, significantly increase morbidity in older adults [23]. More than one-third of adults over 65 years of age suffer cataracts annually, causing 1 in 10 falls with serious injuries [18]. Diabetes is another influential factor in episodes of trauma, especially in falls. Thus, adults with type 2 diabetes mellitus have an increased risk of falls [17]; however, the factors that determine this relationship are not yet known. We can also find evidence that problems related to blood pressure could lead to falls [18,23].

In short, although the literature has highlighted the relationship between the consumption of psychotropic medications and trauma, there is a lack of evidence on the impact that the consequences of the consumption of benzodiazepines, opioids and tricyclic antidepressants (osteoporosis, increased BMI) or other variables independent of the consumption of psychotropic medications (age, gender, cognitive decline, sensory conditions, diabetes, hypertension) may have on this relationship. Additionally, studies that have explored the prevalence of the consumption of benzodiazepines, opioids and tricyclic antidepressants and other substances in trauma patients find a high percentage of falls associated with people who consume these substances [24,25] but do not determine the effect size of the association between these factors or the role that other variables could play in the association. Therefore, the objective of the present study was to determine, in patients who have suffered trauma, the effect size of the association between psychotropic medication consumption and falls by studying the weights that the mediating and confounding variables may have in this association.

## Methodology

A cross-sectional analytical study was based on data obtained from a secondary prevention SBIRT-based program for traumatized patients associated with alcohol and/or other drugs at the Trauma Hospital of Granada. The research was approved by the Granada Provincial Research Ethics Committee (CEI-Granada) and conducted according to the principles expressed in the Declaration of Helsinki; including informed consent obtained from the participants or their relatives or guardians in minor participants.

The population for this study was 1060 patients admitted for trauma in the aforementioned hospital, in the periods in which the programme was active, comprising 34 non-consecutive months during which it received public financial support: from November 2011 to October 2012, June 2013 to November 2013 and June 2014 to February 2015.

### Study variables

The presence of benzodiazepines and analogues (zopiclone, zolpidem and zaleplon), prescription opioids, tricyclic antidepressants and drugs of abuse (cannabis, cocaine, amphetamines, methamphetamines, heroin, and methadone) was detected by urinalysis via fluorescence immunoassay (LifeSign StatusFirst ^®^). Review of the patient’s medical records and direct questioning were used to distinguish between patients who screened positive for positive results for benzodiazepines and opioids as a result of emergency treatment of their injuries and patients who had taken these substances before they sought medical attention. Alcohol was considered positive if the blood alcohol level exceeded 0.3 g/L on admission or if, given the narrow detection window, the patient self-reported alcohol consumption.

Age, gender, presence of a diagnosed psychiatric disorder, mechanism of injury (traffic accident, sports accident, aggression, same-level falls or falls from a height, blows-cuts and other mechanisms), severity of the trauma measured by the injury severity score (ISS) [26], hospital mortality, and variables of exposure to the aforementioned substances were collected prospectively during the implementation of the secondary prevention protocol project. Cognitive impairment prior to trauma; diagnoses of diabetes, osteoporosis, hypertension, heart disease, and obesity; and/or impaired balance, musculoskeletal conditions or sensory system conditions were collected retrospectively during the months of January to May 2016 for this study, using the Medical History of the Andalusian Health System Diraya ^®^ [27]

### Data analysis

For the data analysis, the presence of benzodiazepines, opioids and tricyclic antidepressants was dichotomized as positive or negative. Trauma severity was classified according to the ISS as mild (1–8), moderate (9–15) or severe (≥16). As a dependent variable, the mechanism of injury was used, categorized into 2 strata: same-level falls versus other mechanisms.

For the descriptive study, after verifying the lack of normality in the distribution of the continuous variables, the distribution of these variables was compared using the Mann-Whitney U test. To examine the association of categorical variables, the Pearson Chi-squared test was used.

**Fig 1:**
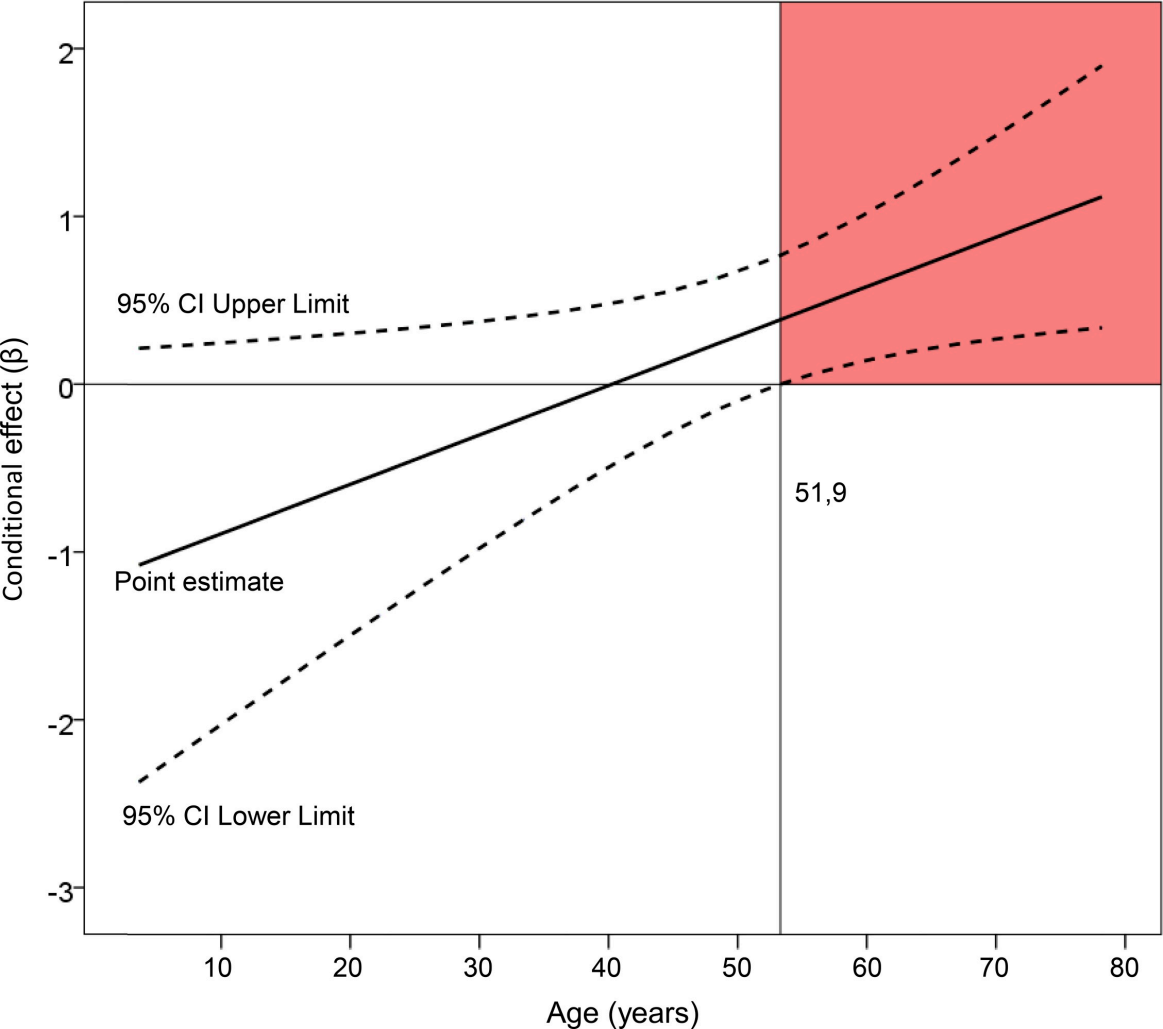
Conditional effect of age in the association between the use of benzodiazepines, opioids and tricyclic antidepressants and falls.

For the analytical analysis, the strength of the association between the consumption of benzodiazepines, opioids and tricyclic antidepressants and falls was quantified by applying a binary logistic regression model where negative outcomes for psychopharmaceuticals and other non-fall injury mechanisms were considered as reference categories. This allowed obtaining crude odds ratios (ORc) and adjusted odds ratios (ORa). For ORa, the independent variables psychiatric disorder, cognitive decline, hypertension, osteoporosis, diabetes, impaired balance, musculoskeletal conditions, heart disease, sensory conditions, obesity, alcohol consumption, cannabis consumption, cocaine consumption, amphetamine consumption and methadone consumption were added to the model. In addition, exploratory analyses were performed to determine which factors acted as effect modifiers. These analyses were developed according to the method of Hayes and colleagues [28,29] through the macro PROCESS in SPSS [29]. This method allowed us to measure the conditional effect of the association between the use of benzodiazepines, opioids and tricyclic antidepressants and falls with which Fig 1 has been carried out. All analyses were performed using SPSS 24.0 for Windows (SPSS, Chicago, IL). The confidence interval was established at 95% (CI 95%), considering values of p ≤ 0.05 significant.

## Results

### Descriptive study

In the comparison of the demographic and clinical characteristics between the groups consuming or not consuming benzodiazepines, opioids and tricyclic antidepressants (Table 1), age was higher in the group of patients consuming benzodiazepines, opioids and tricyclic antidepressants, with a median (interquartile range) of 48 years (39.25–59) versus an age of 42 years (28–53) for patients negative for benzodiazepines, opioids and tricyclic antidepressants. With regard to gender, equality between male and female consumers of benzodiazepines, opioids and tricyclic antidepressants was notable (96 patients, 50%). In the group of patients negative for benzodiazepines, opioids and tricyclic antidepressants, male gender represented 74.1% (643 patients), with female gender representing 25.9% (225 patients). Previous diagnosis of a psychiatric disorder was lower in the group of patients negative for benzodiazepines, opioids and tricyclic antidepressants (1.4%, 12 patients) than in the group positive for these substances (39.1%, 75 patients).

**Table 1:** Baseline characteristics of the groups.

	Non-users of benzodiazepines, opioids and tricyclic antidepressants (n = 868)	Users of benzodiazepines, opioids and tricyclic antidepressants (n = 192)	p value
**Age (years) Median (IQR)**	42 (28–53)	48 (39.25–59)	<0.001
**Sex n (%)**			
Male	643 (74.1)	96 (50)	<0.001
**Psychiatric Disorder n (%)**	12 (1.4)	75 (39.1)	<0.001
**Mechanism of injury n (%)**			
Traffic collision	253 (29.1)	30 (15.6)	
Sports injury	80 (9.2)	6 (3.1)	
Assault	46 (5.3)	12 (6.2)	
Falls on the same level	245 (28.2)	77 (40.1)	<0.001
Falls from a height	105 (12.1)	35 (18.2)	
Cuts or bruises	97 (11.2)	12 (6.2)	
Other mechanisms	42 (4.8)	20 (10.4)	
**Injury Severity Score n (%)**			
Mild: 1 to 8	605 (69.7)	130 (67.7)	
Moderate: 9 to 15	156 (18)	38 (19.8)	0.828
Severe: ≥16	107 (12.3)	24 (12.5)	
**Death n (%)**	18 (2.1)	2(1)	0.342
**Cognitive Impairment n (%)**	6(0.7)	4 (2.1)	0.154
**Diabetes n (%)**	66 (7.6)	16 (8.3)	0.743
**Osteoporosis n (%)**	14 (1.6)	9 (4.7)	0.028
**HBP n (%)**	111(12.8)	35 (18.2)	0.108
**Alterations of Balance n (%)**	5(0.6)	0 (0.0)	0.455
**Locomotor system diseases n (%)**	7(0.8)	6 (3.1)	0.024
**Heart Disease n (%)**	37 (4.3)	9 (4.7)	0.762
**Sensorial Alterations n (%)**	23 (2.6)	6 (3.1)	0.024
**Obesity n (%)**	22 (2.5)	9 (4.7)	0.258
**Other substance detected n (%)**			
Alcohol	196(22.6)	54 (28.1)	0.102
Cannabis	104 (12)	34 (17.7)	0.033
Cocaine	40(4.6)	20 (10.4)	0.002
Anphetamine	1(0.1)	8 (4.2)	<0.001
methadone	0 (0.0)	23 (12)	<0.001

IQR: Interquartile range. HBP: High blood pressure. Benzodiazepines, opioids and tricyclic antidepressants: benzodiazepines and analogues, tricyclic antidepressants, opiate derivatives (excluding heroin and methadone).

We also found differences in the distribution of the mechanism of injury, with a much higher percentage of falls in the group of psychopharmaceutical consumers compared to non-consumers (40.1% vs. 28.2%, *p* < 0.001). Regarding the other variables, we did not find differences in trauma severity; however, there was a higher percentage of osteoporosis in the group positive for benzodiazepines, opioids and tricyclic antidepressants (4.7% versus 1.6% in the non-consumer group). Regarding musculoskeletal deficiency, 3.1% (6 patients) of those who consumed psychopharmaceuticals had some deficiency, compared to 0.8% (7 patients) of non-consumers. Regarding sensory conditions, 3.1% (6 patients) of those who consumed psychopharmaceuticals had some type of condition, compared to 2.6% (23 patients) of non-consumers.

Regarding the consumption of other drugs, significant differences were found, with an increased consumption of other substances (cannabis, cocaine, amphetamines and methadone) in the group positive for psychotropic medicines.

### Analytical study

Table 2 shows estimates of crude and adjusted ORs to quantify the effect size of the association of each variable with same-level falls. If we examine the analysis of the crude association, i.e., of all the variables independently with same-level falls, there is a significant association in almost all, except cognitive decline and methadone consumption. The consumption of benzodiazepines, opioids and tricyclic antidepressants was associated with an ORc (95% CI) of 1.96 (1.40–2.75), *p* < 0.001. However, when all variables are introduced into the multivariate analysis, this association is not observed, leaving only a significant relationship of age and gender with falls, with an ORa (95% CI) of 1.04 (1.03–1.05), *p* < 0.001, and 3.00 (2.16–4.17), *p* < 0.001, respectively. In addition, the relationship between trauma and sensory conditions was also maintained; however, it loses strength, and the consumption of cannabis appears to be a protective factor against sensory condition symptoms.

**Table 2:** Multivariate analysis*. Association between study variables and falls.

	cOR (95%CI)	*p*	aOR (95%CI)	*p*
Age	1.06 (1.05–1.07)	***<0*.*001***	1.04 (1.03–1.05)	***<0*.*001***
Sex (Male Reference)	4.26 (2.21–5.47)	***<0*.*001***	3.00 (2.16–4.17)	***<0*.*001***
Cognitive Impairment	1.53 (0.43–5.48)	*0*.*509*	0.78 (0.19–3.21)	*0*.*734*
Diabetes	2.23 (1.41–3.51)	***0*.*001***	0.91 (0.52–1.57)	*0*.*724*
Osteoporosis	8.68 (3.19–23.6)	***<0*.*001***	1.76 (0.60–5.14)	*0*.*300*
HBP	3.37 (2.35–4.81)	***<0*.*001***	1.52 (0.97–2.39)	*0*.*069*
Alterations of Balance	9.27 (1.03–83.3)	***0*.*047***	6.22 (0.56–68.7)	*0*.*136*
Locomotor system diseases	5.28 (1.61–17.3)	***0*.*006***	2.43 (0.66–8.91)	*0*.*179*
Heart Disease	1.99 (1.10–3.61)	***0*.*024***	0.72 (0.34–1.51)	*0*.*385*
Sensorial Alterations	4.57 (2.10–9.93)	***<0*.*001***	2.54 (1.03–6.28)	***0*.*043***
Obesity	2.20 (1.08–4.52)	***0*.*031***	0.85 (0.38–1.90)	*0*.*693*
Psychiatric Disorder	1.79 (1.14–2.79)	***0*.*011***	0.92 (0.48–1.77)	*0*.*810*
Epilepsy	1.28 (0.42–3.84)	*0*.*663*	1.27 (0.35–4.62)	*0*.*716*
Alcohol detected	0.56 (0.40–0.78)	***0*.*001***	0.92 (0.62–1.35)	*0*.*662*
Cannabis detected	0.15 (0.08–0.29)	***<0*.*001***	0.45 (0.22–0.92)	***0*.*028***
Cocaine detected	0.11 (0.03–0.36)	***<0*.*001***	0.38 (0.11–1.31)	*0*.*125*
Methadone detected	0.34 (0.10–1.14)	*0*.*081*	0.64 (0.08–5.50)	*0*.*687*
Heroine detected	0.36 (0.14–0.93)	***0*.*036***	1.21 (0.22–6.68)	*0*.*824*
**Benzodiazepines, opioids and tricyclic antidepressants detected**	1.96 (1.40–2.75)	***<0*.*001***	1.11 (0.67–1.86)	*0*.*679*

* By binary logistic regression; mechanism of injury other than falls as a reference. 95%CI: 95% confidence interval. Benzodiazepines, opioids and tricyclic antidepressants: benzodiazepines and analogues, tricyclic antidepressants, opiate derivatives (excluding heroin and methadone). Significant P values (< 0.05) are in bold.

In the mediation/moderation analyses, we found that the non-standardized direct effect size of psychotropic medication consumption with falls was β: 0.73 (95% CI: -0.28; 9.09), *p* = 0.066. However, age exerted a significant interaction on the effect of psychotropic medication consumption on falls (p = 0.025), with conditioned effect values that increased with age, with significant values starting at 51.9 years (Fig 1).

## Discussion

When determining the relationship between the consumption of benzodiazepines, opioids and tricyclic antidepressants and falls, assessing the impact of other variables in this association, our main finding is that although the crude association shows a clear relationship between most variables that, a priori, could be linked to this mechanism of injury, this association is not observed after introducing all these factors in a multivariate model. Only the variables age, gender, and sensory conditions remain significantly associated, and negatively so with cannabis consumption. The study of mediation/moderation showed that of these variables, age modified the effect of benzodiazepines, opioids and tricyclic antidepressants in falls, becoming significant after 51.9 years of age. These results suggest that the consumption of benzodiazepines, opioids and tricyclic antidepressants is not directly related to a greater number of falls compared to other mechanisms of injury in trauma patients, as noted by some studies [24], but the effect of the association of the consumption of these substances with the falls is progressively conditioned by age; with a significant conditional effect from 51.9 years. On the other hand, falls appear associated with female gender and, to a lesser extent, sensory conditions (blindness, deafness, etc.). The emergence in our study of cannabis as an inversely associated factor with the consumption of falls could be due to the known strong link existing in the consumption of this substance with other mechanisms, such as traffic accidents [30].

Numerous studies have confirmed the association between psychotropic medication use and falls in elderly people [15,16,31–33]. Pratt et al 2014 [15] found an increased numbers or increased doses of psychoactive medicines are associated with an increased risk of hospitalization for falls in older adults with adjusted models by age and sex. However, important methodological differences prevent us from comparing our results: First, the study by Pratt et al 2014 was carried out only with a population over 65 years of age; by decreasing the detection spectrum by age it is more difficult to determine its influence on the association. In addition, although the study has a considerable sample size, due the use of records to determine the exposure, it is possible that some patients who were classified as being exposed only took the medicine for a few days or did not consume the medicine, similarly, patients who took the drug without a prescription could be mistakenly classified as non-consumers. To the best of our knowledge, no previous study has investigated the possible influence of mediating and confounding variables in the relationship between psychotropic medication consumption and falls in trauma patients by prospective systematic screening of exposure to these substances. Therefore, we believe that this study provides a novel point of view for understanding the factors that are related to trauma incidents and their characteristics.

Our descriptive study shows that the age of consumers of benzodiazepines, opioids and tricyclic antidepressants that have suffered trauma, average of 48 years of age, is clearly higher than that of the rest of the patients, a finding that coincides with those of the Spanish Monitoring Centre for Drugs and Drug Addiction (Observatorio Español de la Droga y las Toxicomanías) and other studies that place the age of onset of the use of these substances at 45 years of age, much higher than that of other substances of abuse [9,34,35]. For gender, although traumatic pathology is strongly linked to male gender [36], the percentage of women who consumed psychopharmaceuticals in our study was similar to that of men, a reflection of the strong association that exists between the consumption of these substances and the female gender in the general population [9,19].

In line with the findings of the Report on Drug Use in Spain, published in 2015 [34], our data also indicate that alcohol is the most frequently consumed substance. In fact, alcohol consumption in Spain is considered universal because more than 93% of people aged 15 to 64 consume alcoholic beverages on occasion [34], and the association between consumption and trauma is widely known [37]. Surprisingly, benzodiazepines were the second most common substance found after cannabis, which supports the increasing trend in our country of the consumption of these drugs, with or without a prescription [38], observing a growing trend since 2005 in the consumption of hypnosedatives; however, the perceived risk has not increased [34]. This fact, coupled with increasing evidence indicating an increased risk of trauma associated with these substances [13], should provide a warning for our country, especially for older individuals who consume these substances because falls are a frequent problem in people over 65 years of age, and the consumption of these medications is directly related to an increased risk of falls [39]. Some studies report a decrease in falls by up to 66% through minimizing prescriptions for these substances [40].

Notably, in our sample, there was a significantly higher number of drugs other than alcohol in psychotropic medication users. The use of central nervous system depressants to reduce the effect of stimulants such as cocaine and methamphetamine has also been found in previous studies [41].

The main limitations of this study include the impossibility of extrapolating the results to patients who suffer trauma and are not hospitalized. Furthermore, the retrospective collection of some of the variables used in the study, through a review of patient medical records, could lead to underestimations of some pathologies, such as those that are still latent and have not been diagnosed, for example, obesity, whose detection sensitivity through this method is questionable. On the other hand, patients who were admitted with blood alcohol levels of 0.3 g/L or lower have not been considered positive for alcohol. This is because it is the legal limit allowed in our country for novice and professional drivers. However, the concentration of alcohol in the blood related to an increased risk of trauma in the elderly may not coincide with that legally established for driving due to the special susceptibility of this age group [42].

Despite these limitations, this study presents important strengths. It is noteworthy that this study was carried out within a project in which a systematic screening of substances in patients admitted for trauma was carried out. In this study, positive tests for benzodiazepines and opioids were prospectively excluded when these drugs were administered as part of the prehospital or emergency treatment, thus avoiding false positives. Although there may be some cases that the use of benzodiazepines in prehospital care for serious trauma could have masked the previous consumption of non-prescribed benzodiazepines (if the patient had medical records of prescribed benzodiazepines was screened as positive), we believe that our approach is more adequate than systematically discarding these benzodiazepine positive screenings. On the other hand, the design of the present study allowed us to avoid selection biases present in other studies in which consumption was detected by means of questionnaires [43] or through prescription databases [10]. However, the method employed cannot guarantee the total absence of other potential biases, such as those that could occur due, for example, to trauma associated with drug withdrawal (in which levels may be non-existent or quite low), to exclusion of cases positive for opioids and benzodiazepines (without further efforts to identify pre-trauma use of these commonly used and abused substances), and to episodic or binge use of psychotropic substances can that can produce brain and heart lesions that can precipitate falls and other forms of trauma (especially opioids used with or without benzodiazepines) in the absence of current psychotropic use.

In conclusion, we can affirm that despite the crude data suggesting a relationship between the consumption of benzodiazepines, opioids and tricyclic antidepressants and falls in patients who have suffered trauma, this relationship is influenced by other variables that confound or modify the effect of this relationship. However, the conditional effect that age exerts on this association only begins to be significant after 51.9 years of age. The influence of benzodiazepines, opioids and tricyclic antidepressants on trauma risk cannot be called into question given the methodology and population used in this study; however, future research conducted in this regard should account for the importance of non-causal associations that could be generated if the roles of the variables used in this study are not considered.

## Supporting information

S1 Data File (SAV)
